# Prevalence of Scoliosis in Children and Adolescents with Cystic Fibrosis

**DOI:** 10.3390/children11030321

**Published:** 2024-03-08

**Authors:** Valentina Fainardi, Monica Nora, Annamaria Salghetti, Federica Petraglia, Patrizia Fanzaghi, Giulia Anelli, Marta Cattabiani, Giuseppe Scopelliti, Michela Deolmi, Ilaria Ferrari, Francesco Longo, Susanna Esposito, Giovanna Pisi

**Affiliations:** 1 Cystic Fibrosis Unit, Pediatric Clinic, Department of Medicine and Surgery, University of Parma, 43126 Parma, Italygpisi@ao.pr.it (G.P.); 2 Rehabilitation Medicine Service, Rehabilitation Geriatrics Department of the University Hospital of Parma, 43126 Parma, Italy; mnora@ao.pr.it (M.N.); fpetraglia@ao.pr.it (F.P.); ganelli@ao.pr.it (G.A.); mcattabiani@ao.pr.it (M.C.); 3 Respiratory Disease and Lung Function Unit, Department of Medicine and Surgery, Hospital of Parma, 43126 Parma, Italy

**Keywords:** scoliosis, spinal deformity, children, cystic fibrosis, bone disease

## Abstract

The prevalence of scoliosis in people with cystic fibrosis (CF) seems to be greater than in the normal population. Over the last two years, a screening for spinal deformities was carried out in patients with CF aged 5 to 18 years, followed up at the CF regional Centre in Parma (Italy). Forty-three patients (twenty-seven males, mean age: 11.8 ± 4.5 years) were enrolled in the study. Nine patients (20.9%) were diagnosed with scoliosis, with a mean Cobb angle of 20.8 ± 9.4 (12–38°). Five patients (11.6%) were diagnosed with a postural kyphosis attitude and one with pathological fixed kyphosis. All patients with scoliosis and postural kyphosis started daily physiotherapeutic scoliosis-specific exercises (PSSE). Compared to people without CF, the prevalence of scoliosis in our paediatric CF population seems to be higher and more present in males; the curves were thoracic and mostly right-sided. CF disease, hyposthenic postural attitude and sedentary lifestyle can contribute to the pathogenesis of this musculoskeletal alteration. Spinal deformities may negatively affect pulmonary function, resulting in disability, pain and a decreased quality of life. Since the prevention of musculoskeletal deformities is easier than restoration, in CF population targeted screening during growth and interventions, including regular physical exercise, are mandatory.

## 1. Introduction

Cystic fibrosis (CF) is the most common autosomal recessive genetic disease and is caused by mutations in the gene encoding the cystic fibrosis transmembrane conductance regulator (CFTR) protein, which leads to reduced CFTR quantity and function in the respiratory tract, pancreas, gastrointestinal system and sweat glands. In the lungs, thickened mucus impairs mucociliary clearance and leads to frequent respiratory infections. In the pancreas, thickened secretions obstruct the intra-pancreatic ducts, reducing the release of digestive enzymes into the intestine and resulting in the malabsorption of fat and fat-soluble vitamins [1,2]. Malnutrition and poor growth are common issues among children with CF [3,4] and are strongly associated with poor lung function, exacerbations and survival [5,6].

A further comorbidity of CF is bone disease, for which malnutrition and recurrent pulmonary infections are well-known risk factors [7]. In individuals with CF, skeletal manifestations have been widely reported, including diminished mineral bone density and content with high rates of osteopenia, osteoporosis and fractures, and postural deviations such as scoliosis and hyperkyphosis [8,9,10,11,12,13,14,15]. Scoliosis is a general term comprising a heterogeneous group of conditions and has been described as a torsional deformity of the spine. The spinal deformity caused by idiopathic scoliosis (IS) may be defined as a sign of a syndrome with a multifactorial aetiology [16]. Many different classifications of IS have been proposed: chronological classification, based on the age of the child at which the deformity is diagnosed [infantile (0–2 years), juvenile (3–9 years), adolescent (10–17 years) or adult (18+)]; topographic classification, based on the anatomical site of the apex of the curve in the frontal plane (cervical, apex C6–C7; cervicothoracic, apex between C7 and T1; thoracic, apex between T1–T2 and T11–T12; thoracolumbar, apex between T12 and L1; lumbar, apex L1–L2); and angular classification, based on the angle measured on a standing frontal radiograph according to the Cobb method. Scoliosis can be diagnosed when the Cobb angle is greater than 10°. The chronological classification is important in quantifying the risk of developing a more severe and complicated deformity, while the angular classification is crucial in the management of IS since it is directly correlated with treatment decisions [16,17,18]. Scoliosis in CF has been previously investigated [11,14,19,20,21,22], and most authors have found that in these patients its prevalence is higher compared to the healthy population, where the prevalence ranges from 0.2 to 2.2% with a preference for girls, especially in older age and when the curve is higher [23,24]. On the other hand, kyphosis is an excessive curvature of the thoracic spine in the standing position. As for scoliosis, the kyphosis angle can be measured by the Cobb angle from an examination of radiographic imaging in the sagittal projection. Some risk factors for kyphosis are vertebral fractures, decreased muscle strength and degenerative changes in the spine. Kyphosis can be congenital, postural or associated with neuromuscular diseases or skeletal dysplasia. The commonest type of kyphosis is postural kyphosis, where the spine remains flexible and the curvature can be corrected with improved posture and specific exercises [25]. In patients with chronic respiratory disease, both scoliosis and kyphosis can affect pulmonary function and worsen respiratory symptoms [26,27].

The aim of this study was to assess the prevalence of scoliosis and other spinal deformities in our cohort of children and adolescents with CF.

## 2. Methods

Between 2021 and 2023, all children with CF aged 5–18 years attending Parma CF Centre (Italy) were screened for spinal deformities, including scoliosis and kyphosis, by three physiatrists (AS, FP, MN) with experience in clinical practice approaches to IS during growth.

The assessment was based on evidence-based clinical guidelines for postural evaluations on the frontal plane and sagittal profile [e.g., Adam’s forward bending test, Angle of Trunk Rotation (ATR) measured with a scoliometer, plumbline imbalance]. The intra- and interrater reliability of the ATR was excellent and very good, respectively [28]; a good correlation between the scoliometer measurements and radiograph analyses was demonstrated [28,29]. In the case of clinical suspicion, X-rays of the spine barefoot and in standing position (PA and LL view) were performed, and the Cobb angle was measured by physiatrists. Scoliosis was defined as a Cobb angle of more than 10° in AP projection and a curvature classified as cervical (apex between C2 and C6), cervicothoracic (apex between C7 and T1), thoracic (apex between T2 and T11) or thoracolumbar (apex between T12 and L1); the side of convexity was also recorded. Patients were then treated according to the literature [30,31,32,33]. The diagnosis of kyphosis was made by clinical examination and, when needed, X-rays of the spine barefoot and in standing position (LL view); postural kyphosis has been defined as a non-structural but reversible kyphosis [25].

Demographic data [(age, sex, body mass index (BMI)], quality of life (QoL), lung function (mean value of the last 4 measurements) and the number of exacerbations requiring antibiotics in the previous 12 months before scoliosis diagnosis were collected. QoL was assessed with the CF questionnaire—revised (CFQ-R) [34]. Global Lung Function Initiative (GLI) 2012 reference equations were used to determine the percent of predicted forced expiratory volume in the 1st second (FEV_1_) based on age, sex, height and ethnicity [35]. Pulmonary exacerbations were defined as a clinical worsening and/or increase in productive cough requiring oral or intravenous antibiotics or hospitalisation.

This study was performed in accordance with the Declaration of Helsinki. This human study was approved by the Ethics Committee of the Az. Ospedaliero-Universitaria di Parma (921/2021/OSS*/AOUPR, 25 May 2022). All participants and parents provided written informed consent to participate in this study.

### Statistical Methods

Normally distributed variables were reported as a mean and SD, and non-normally distributed variables were reported as a median and range. For categorical and dichotomous variables, differences between groups were tested by using the chi-square test or Fisher test; for quantitative variables, differences between groups were tested by a *t*-test. A two-tailed *p*-value of <0.05 was considered statistically significant. The data analysis was performed using SPSS version 26 (IBM Corp. Released 2019. IBM SPSS Statistics for Windows, Version 26.0. Armonk, NY: IBM Corp).

## 3. Results

Forty-three patients (twenty-seven males; mean age: 11.8 ± 4.5 years; BMI: 18.3 ± 3.3) were included in the study.

Nine patients (20.9%) were diagnosed with scoliosis (six males; 14.3 ± 2.7 years; BMI: 19.9 ± 3.2), with a mean Cobb angle of 20.8 ± 9.4 (12–38°). Three patients had a curve >20°, and three had a double curve. Of the patients with a single curve, five out of six had a right-sided curve. All curves were thoracic, with five also involving the first part of the lumbar spine.

Although not significant, the patients with scoliosis seemed to have lower lung function compared to the rest of the population (FEV_1_% pred: 79.6 ± 18.7% vs. 93.9 ± 20.9%, *p* 0.069; FVC% pred: 87.9 ± 14.5 vs. 94.1 ± 19, *p* 0.374; FEV_1_/FVC: 0.90 ± 0.15 vs. 0.99 ± 0.10, *p* 0.051), with more than one-third showing airway obstruction (i.e., FEV_1_ < 80%) (33% vs. 15%).

The walking distance test (predicted values) was similar between patients with scoliosis and the patients without scoliosis (80.8 ± 6.6% vs. 83.6 ± 10.7%, *p* 0.487).

Six patients (11.6%) were diagnosed with postural kyphosis (five males; 16.3 ± 2.2 years; BMI: 20.3 ± 2.2). The patients with scoliosis had a lower lung function compared to those with postural kyphosis (FEV_1_% pred: 79.6 ± 18.7% vs. 114.1 ± 17.2, *p* 0.020).

All the patients with scoliosis and postural kyphosis started daily physiotherapeutic scoliosis-specific exercises (PSSE); the three patients with a scoliotic curve >20° were treated with a brace. Table 1 describes the characteristics of the patients with scoliosis and kyphosis, or a postural kyphosis attitude.

Overall, the patients with spinal deformities (scoliosis and postural kyphosis) were older and had higher BMIs than the patients without scoliosis or postural kyphosis. No differences were noted in lung function, exacerbation rate or QoL scores. Table 2 describes the children and adolescents with spinal deformities (scoliosis and postural kyphosis) compared to the rest of the patients.

## 4. Discussion

In our cohort of patients with CF, we found a higher prevalence of scoliosis compared to the prevalence reported in large population studies conducted on people without CF (20% vs. 0.02–2.2%) [23,24]. The curves were mainly right-sided and thoracic, confirming the data reported in previous works [11,14,19,22]. All the curves were considered idiopathic. Although FEV_1_% pred and FEV_1_/FVC seemed to be lower in patients with scoliosis, these differences were not significant (FEV_1_% pred: 79.6 ± 18.7% vs. 93.9 ± 20.9%, *p* 0.069; FEV_1_/FVC: 0.90 ± 0.15 vs. 0.99 ± 0.10, *p* 0.051). In addition, six patients had postural kyphosis.

In a recent epidemiologic study conducted in Italy, a screening program for scoliosis found a prevalence of scoliosis of 0.76% (65 of 8995 children) in children aged 9–14 years with a ratio of boys to girls of 1:3, reaching 1:4 when the curves had a Cobb angle ≥30°. The curves were mainly double and thoracolumbar [24]. Previous studies have already noted an increased prevalence of scoliosis in children with CF, and all reported that thoracic right-sided curves were the most frequent types. The first published study reported an overall prevalence of 5% in 203 CF patients (aged 3 months–32 years), increasing to 11.9% in adolescents over 15 years of age [20]. Paling and co-workers observed a prevalence of 10% in 151 patients, including children over 4 years of age and adults, with a majority of thoracic right-sided curves but no gender predilection [19]. In a sample of 90 children aged 4–16 years, the scoliosis prevalence was 15.6%; most of the children were girls, and the curve was thoracic with no gender predilection [14]. Another group from the UK analysed 143 CF subjects with an age range of 10–18 years and found that 16 had scoliosis (a prevalence of 11%) with a median Cobb angle of 14° (range 10–38); all of them had thoracic curves [22]. In 51 Turkish patients with CF (aged 6–28 years), the prevalence of scoliosis was 17% [36]. In contrast with these studies, the prevalence reported in a cohort of patients followed up at Royal Brompton Hospital (UK) (aged 1–18 years) was lower (2.2%) and within the range for the general paediatric population; all curves were thoracic and right-sided [11].

IS is usually a benign condition, but, compared to a healthy population, in CF patients, it may have a different magnitude. In patients with chronic respiratory conditions, scoliosis may alter the shape of the chest, worsen pulmonary restriction and lead to reduced lung capacity. Scoliosis or other spinal deformities can decrease the mobility of the thoracic cage and spine during breathing, which in people with CF can further worsen respiratory symptoms like dyspnoea or cough [26,27]. As a result, patients with obstructive respiratory disease with these skeletal issues may complain of muscle contractures and postural abnormalities [26] with pain in the back and trunk [37,38], which can interfere with daily treatments like airway clearance therapy and physical exercise [39].

The higher prevalence of scoliosis found in patients with CF raises the question of whether this type of scoliosis is different from idiopathic-type curves and whether other predisposing factors like malnutrition, disease severity or physical inactivity can influence the development of a spinal deformity.

Malnutrition due to the malabsorption of nutrients and fat-soluble vitamins like vitamin D is a common finding in children with CF, leading to poor growth, reduced BMI and decreased bone mineral density [6,40]. In addition, less sun exposure, chronic inflammation developing from lung infections, physical inactivity and endocrinological disorders like delayed puberty can also have a role in bone disease [41]. CF-related bone disease (CFBD) is defined as the presence of a low bone mineral density based on dual-energy X-ray absorptiometry (DXA). A meta-analysis of multiple case series reported that the pooled incidence of osteoporosis in people with CF was 23.5%, and the pooled incidence of osteopenia was 38% [8]. However, 40% of adult patients with CF do not have a DXA completed [42], therefore limiting the reliability of the data and suggesting a possible higher rate of bone disease in this population. Osteoporosis and osteopenia can be associated with a higher risk of spinal deformities like thoracic kyphosis and scoliosis, rib and spinal fractures and vertebral wedging. These alterations can reduce respiratory function, impair airway clearance and cause chronic pain [7,8]. Only one study on the prevalence of scoliosis in CF assessed CFBD, reporting that children and adolescents with scoliosis generally had poorer bone mineral density, although no correlation was demonstrated with scoliosis severity [14]. Malnutrition correlates with disease severity and exacerbations [5,6], which might be further risk factors for the development of spinal deformities. During exacerbations, often a patient with CF assumes a forward-leaning posture to facilitate breathing through the recruitment of respiratory accessory muscles. This effort is associated with the overuse of respiratory muscles, which may increase trunk stiffness and soft tissue contractures, facilitating postural abnormalities.

Malnutrition, bone disease and respiratory exacerbations may prevent people from regularly engaging in physical activity. Smaller muscle size and impaired muscle function have been reported in subjects with CF [43,44] but also in adolescents with IS [45], suggesting that both physical inactivity and scoliosis can contribute to the reduction in exercise capacity of CF patients. On the other hand, regular moderate-to-vigorous physical activity has been associated with a 30% decreased risk of developing scoliosis [46]. Subjects without CF but with IS practice less physical activity and spend more time carrying out sedentary activities [47]. A sedentary lifestyle has been found to affect bone health in healthy adolescents [48]. Interestingly, in healthy children, prolonged relaxed sitting increases trunk asymmetry [49], and in adolescents with IS, it contributes to a worsening of scoliosis due to habitual leaning on the concave side of the curve [50]. The promotion of regular physical activity and reduction in sedentary habits can be crucial to slowing down the progression of scoliosis curvature, increasing trunk mobility and muscle strength [45], and therefore protecting respiratory function in patients with chronic respiratory conditions [27,51]. Furthermore, regular physical activity has been associated with improvements in bone mineral density.

**Figure 1:**
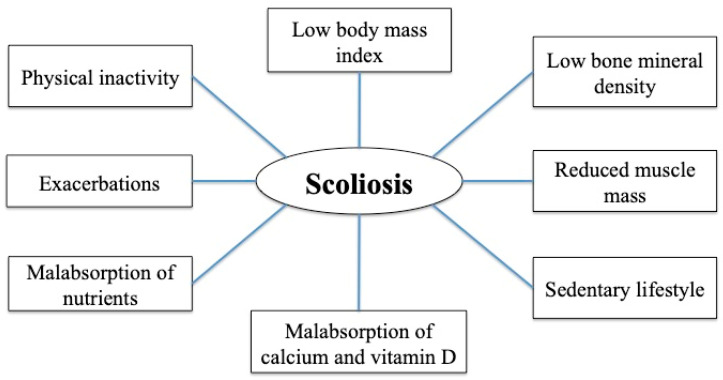
Multifactorial origin of scoliosis in people with cystic fibrosis.

In our study, the patients with spinal deformities had normal BMIs, discouraging the hypothesis that malnutrition could be a risk factor for the development of scoliosis or kyphosis. However, the children and adolescents with a diagnosis of scoliosis seemed to have lower values for FEV_1_, suggesting that this condition may contribute to lung function impairment and be a further comorbidity of those patients with more severe disease. Similarly, the older age of patients with spinal deformities (15 years vs. 10 years) may suggest a more advanced stage of the disease. The higher prevalence of scoliosis found in our cohort of CF patients might have been facilitated by hyposthenic postural attitudes, reduced muscle tone and a sedentary lifestyle, as reported in the literature [43,44,47,50]. However, we can only speculate about the multifactorial origin of scoliosis because the small numbers prevent us from drawing firm conclusions. Figure 1 describes the possible factors affecting scoliosis development in CF patients.

**Figure 2:**
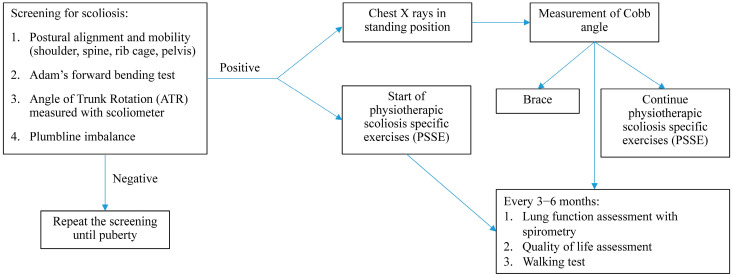
Proposed approach to investigating spinal deformities in young patients with cystic fibrosis.

In our cohort of patients, we also found that some had postural kyphosis. Postural kyphosis is a non-structural curvature of the thoracic spine originating from poor posture during adolescence; it is usually reversible with specific exercises. Patients with CF exert repetitive positive pressure on the thorax when coughing. This causes prolonged outward pressures that can result in thoracic kyphosis, urinary stress incontinence, protraction of the scapulae and lumbar pain. In addition, the prolonged use of a trunk flexion posture for coughing may further increase the risk of thoracic hyperkyphosis [52]. In a Turkish population of CF patients over 6 years of age, the prevalence of thoracic kyphosis was higher compared to a healthy population (5.1% vs. 1%) [36]. In 41 Polish children and adolescents with CF, 43% had increased thoracic kyphosis [53]. In a recent study performed on 28 Brazilian CF patients, thoracic kyphosis was negatively correlated with FEV_1_ and the time spent carrying out moderate-to-vigorous physical activity, suggesting the negative impact of this spinal deformity on lung function and exercise [54]. In our cohort, the kyphosis was still postural and therefore reversible, and lung function was within the normal range. On the contrary, the patients with scoliosis showed significant lower FEV_1_ values, maybe due to a structural deformity of the spine. Data on older patients with postural kyphosis during adolescence are needed to evaluate the evolution of this condition and its effect on lung function. The early detection and treatment of spinal deformities in people with CF might be crucial for the early prevention of late-onset musculoskeletal complications that may interfere with QoL, daily physical activity and disease progression. Approximately 10% of IS patients require conservative treatment, and approximately 0.1–0.3% require operative correction [55]. Progression is more common in girls during growth spurts in puberty, especially when the Cobb angle is greater than 20° [55]. According to current evidence, IS non-operative treatments include PSSE [56,57] and/or bracing [58], depending on curve severity and progression [59]. Conservative treatment with rigid bracing and PSSE is recommended for Cobb curve angles above 20–25° in skeletally immature patients. Bracing treatment involves wearing a corrective orthosis for a specified amount of time each day, usually until maturity [55]. In our cohort of patients, all those detected with spinal deformities were treated with PSSE, and those with scoliosis and more severe curves were treated with a brace. Specific and targeted physical therapy programmes can decrease respiratory and postural workload, strengthen the thoracic muscles and promote airway clearance. Furthermore, physical wellbeing and muscle strength can encourage the participation of patients in physical activity, which is very important not only from a respiratory point of view but also in terms of socialisation and QoL. However, an open and confident relationship with the patient is mandatory to obtain satisfactory results and adherence to treatments. The patient must be motivated and know what the goal of the treatment is. Nowadays, patients with chronic diseases cannot follow the ‘one size fits all’ approach; instead, each therapeutic programme must be carefully designed around the individual after a comprehensive evaluation. Figure 2 depicts our proposed approach to investigating spinal deformities in young patients with CF.

The limitations of our study are the small number of subjects analysed, the retrospective nature of the study, which limits follow-up assessments, the lack of data on bone disease and the restriction to an age group between 5 and 18 years. In addition, the lack of a control group prevents us from making comparisons between the CF patients and healthy subjects. To overcome this latter limitation, we considered for comparison the data on scoliosis prevalence reported from large epidemiological studies conducted on healthy subjects of the same age group.

Further data are needed on the multifactorial origins of spinal deformities in CF patients and on the efficacy of PSSE and/or braces in this cohort of patients in terms of lung function and quality of life.

## 5. Conclusions

The prevalence of postural deviations in CF patients is higher compared to the rest of the population, with a specific increased risk of scoliosis and kyphosis. Spinal deformities may negatively affect pulmonary function, resulting in disability, pain, reduced levels of physical activity and decreased QoL. Since the prevention of musculoskeletal deformities is easier than restoration, targeted screening during growth and interventions, including regular and specific physical exercises, are mandatory. The optimal time to screen and attempt to prevent musculoskeletal deformities in at-risk subjects is during the pre-pubescent years, around 8–12 years old. The detection and early, appropriate treatment of spinal deformities can improve mechanical support, leading to better posture, reduced back pain, improved sense of well-being and decreased excursion breathing. In the era of modulators, where patients are supposed to be healthier and live longer, more data on scoliosis evolution and its influence on lung function and QoL are needed to promote screening programmes and find preventive measures.

## Figures and Tables

**Table 1:** Characteristics of children and adolescents with scoliosis and postural kyphosis.

	Patients with Scoliosis (*n* = 9)	Patients with Postural Kyphosis (*n* = 6)	*p*
**Age, yrs**	14.3 ± 2.7	16.3 ± 2.2	0.166
**Sex**	6 M (66.6%)	5 M (83.3%)	0.474
**BMI**	19.9 ± 3.2	20.3 ± 2.2	0.791
**Genetics, *n* (%)**	4 delF508/delF508 (44.4%) 2 delF508/other (22.2%) 3 Other (33.4%)	3 delF508/delF508 (50%) 3 delF508/other (50%)	0.830
**Pancreatic insufficiency, *n* (%)**	5 (55.5%)	4 (66.6%)	0.666
**Diabetes, *n* (%)**	3 (33.3%)	2 (33.3%)	0.576
**Colonisation, *n* (%)**	4 *Staphylococcus aureus* 3 *Pseudomonas aeruginosa* 1 *Burkholderia gladioli* 1 Free	5 *Staphylococcus aureus* 1 *Pseudomonas aeruginosa*	0.634
**FEV_1_% pred**	79.6 ± 18.7	114.1 ± 17.2	0.020
**FVC% pred**	87.9 ± 14.5	110.7 ± 12.2	0.101
**FEV_1_/FVC**	0.90 ± 0.15	1.0 ± 0.0	0.139
**Walking test, %**	80.8 ± 6.6	86.9 ± 5.8	0.380
**Exacerbations in the last 12 months**	1 (0–7)	2 (0–7)	0.815
**Hospital admission in the last 12 months**	0 (0–1)	0 (0–1)	0.432
**CFQR**	89.1 ± 14.7	75.9 ± 6.3	0.175

BMI, body mass index; FEV_1_, forced expiratory volume in the 1st second; FVC, forced vital capacity; CFQR, cystic fibrosis questionnaire revised.

**Table 2:** Characteristics of children and adolescents with spinal deformities (scoliosis and postural kyphosis) compared to the rest of the patients.

	Patients with Spinal Deformities (*n* = 15)	Patients without Spinal Deformities (*n* = 28)	*p*
**Age, yrs**	15.1 ± 2.6	10 ± 4.1	<0.0001
**Sex**	11 M (73.3%)	16 M (57.1%)	0.333
**BMI**	20.0 ± 2.7	17.4 ± 3.2	0.011
**Genetics, *n* (%)**	7 delF508/delF508 (46.6%) 5 delF508/other (33.4%) 3 Other (20%)	1 delF508/delF508 (3.5%) 19 delF508/other (68%) 8 Other (28.5%)	<0.005
**Pancreatic insufficiency, *n* (%)**	9 (60%)	11 (39%)	0.194
**Diabetes, *n* (%)**	5 (33.3%)	2 (7.1%)	0.744
**Colonisation, *n* (%)**	4 *Pseudomonas aeruginosa* 2 Free	9 *Pseudomonas aeruginosa* 11 Free	0.350
**FEV_1_% pred**	93.4 ± 24.7	89.6 ± 19.3	0.583
**FVC% pred**	96.1 ± 17.4	91 ± 18.6	0.407
**FEV_1_/FVC**	0.94 ± 0.1	0.98 ± 0.1	0.343
**Walking test, %**	84.3 ± 7.9	82.1 ± 11.1	0.526
**Exacerbations in the last 12 months**	1 (0–7)	1 (0–8)	0.439
**Hospital admission in the last 12 months**	0 (0–1)	0 (0–1)	0.865
**CFQR**	85.5 ± 14	87.7 ± 10.8	0.692

BMI, body mass index; FEV_1_, forced expiratory volume in the 1st second; FVC, forced vital capacity; CFQR, cystic fibrosis questionnaire revised.

## Data Availability

The data presented in this study are available on request from the corresponding author. The data are not publicly available due to privacy considerations.
